# Subcapsular Orchiectomy in the Primary Therapy of Patients with Bone Metastasis in Advanced Prostate Cancer: An Anachronistic Intervention?

**DOI:** 10.1155/2012/190624

**Published:** 2011-09-14

**Authors:** Oleg Rud, Julia Peter, Reza Kheyri, Christian Gilfrich, Ali M. Ahmed, Wieland Boeckmann, Paul G. Fabricius, Matthias May

**Affiliations:** ^1^Department of Urology, St. Elisabeth Klinikum Straubing, St. Elisabeth Straße 23, 94315 Straubing, Germany; ^2^Department of Urology, Vivantes-Klinikum Berlin-Neukölln, 12351 Berlin, Germany

## Abstract

*Background*. The therapeutic impact of palliative androgen deprivation in metastatic prostate cancer is indisputable. Bilateral orchiectomy represents the traditional method of AD but was reduced during the last years in favor for treatment with LHRH analogues. Due to limited economic resources of the health care system, the economically priced definite surgical castration might experience a renaissance. *Methods*. In this single-center retrospective study, 83 consecutive patients with osseous metastasized prostate cancer were evaluated, who had primarily been treated by subcapsular bilateral orchiectomy. Response to therapy, time until therapy failure, overall survival time, psychological disorders due to loss of organ, and disease-associated and postoperative surgical complications were recorded. The median followup was 35 months (IQR: 26–46). *Results*. Patients' mean age at surgery was 72.1 (54–91) years. Six patients (7.2%) displayed immediate tumor progression after orchiectomy. Median time of tumor remission and overall survival time were 29 and 36 months, respectively. 14% of the study group showed minor postoperative complications. No psychological problems occurred following bilateral orchiectomy. *Conclusion*. Due to an effective and persistent oncological effectiveness, less morbidity, and absence of psychological implications, bilateral subcapsular orchiectomy seems to be a practicable and advisable alternative in the first-line therapy of metastasized PCa.

## 1. Introduction

In the pre-PSA era, one third of men with prostate cancer (PCa) presented with distant metastasis at time of diagnosis, and currently, it is about 5%–10% [1]. Despite these changes, PCa still represents the second most frequent tumor-associated cause of death. In 2006, 12000 men died from PCa in Germany [1].

Suppression of endocrine testicular function still represents the gold standard in palliative treatment in advanced stage or metastasized PCa. Already in 1941, Huggins and Hodges demonstrated control of PCa growth rate by androgens and showed that there is no better way to achieve temporary control of PCa growth than androgen deprivation (AD) [2]. Basically, AD treatment is able to induce a remission in 90% of PCa patients; the median progression-free survival ranges from 18 to 34 months [3].

The earliest method of AD is represented by the bilateral orchiectomy, which means a definitive therapy for the patient. Treatment with Diethylstilbestrol (DES) was described as to the first method of reversible castration. At present, medicinal castration is achieved either by LHRH analogues, which have been available since the 1980s or by GnRH antagonists being approved at the end of the last decade. The use of antiandrogens remains the limitation of initial increase of testosterone (flare phenomenon) under treatment with LHRH analogues. Alternatively, they can also be used as monotherapy, especially in patients with marginal metastatic load with consideration of a better quality of life but only slightly shorter progression-free and overall survival in comparison to castration [4]. 

Despite the fact that bilateral orchiectomy represents a proven method showing excellent oncological efficiency with rapid onset of action, 100% compliance of surgical castration due to the definite character, and just minimal side effects, at present, priority is given to medical treatment [5]. One rational explanation for the avoidance of surgical castration might be the expectation of psychological consequences due to loss of testicles [6]. In 1942, Riba described subcapsular bilateral orchiectomy (OE-R) as a surgical method of avoidance of the “empty scrotum” without damage of the oncological effectiveness [7]. Furthermore, American and European studies could clearly prove that medical castration by LHRH analogues is considerably more expensive than surgical castration [8, 9]. 

In the present retrospective study, 83 patients with an osseous metastasized adenocarcinoma of the prostate undergoing OE-R in an 11-year period were analyzed with respect to progression-free and overall survival and discussed against the background of internationally available data on this topic. Furthermore, disease-related and postoperative surgical complications as well as psychological implications were evaluated and discussed.

## 2. Methods

In the time period between January 1990 and December 2000, OE-R was carried out in 98 patients with a metastasized PCa in the clinical center Berlin-Moabit. Based on electronic patient files of the hospital and from urologists in practice, clinical and oncological parameters of 83 patients with bone metastasis, out of whom 63% presented with multiple metastases, could be evaluated. In two out of 15 patients excluded, examination criteria were not completely documented, and in further 13 patients, the OE-R was not accomplished as primary therapy for osseous metastasized PCa. Accurate assessment of PSA kinetics (initial value and nadir) could be evaluated in 67 patients. Patients were offered OE-R in local anesthesia, and surgery was carried out according to the originally published surgical method [7]. Each patient received antibiotic single-shot application at the day of surgery. In all cases, postoperative pain could be managed with nonsteroidal anti-inflammatory drugs. 

Oncologic course of disease was calculated by Kaplan-Meier method according to time of tumor remission and overall survival starting at the moment of OE-R. For the determination of the time until tumor remission, patients who died without tumor progression were censored by time of death. In 16 patients, preoperative PSA values were not known; however, following PSA values were assessed so that even in these patients the end of hormonal sensitivity could be defined by an increase of the PSA value and/or by newly established symptoms. The median followup was 35 months (IQR: 24–26). 

Psychologically relevant disorders of the patient caused by loss of testicles were considered when information of psychological problems was indicated by the patient himself (criteria 1), in case of comedication with antidepressant drugs (criteria 2), or cotreatment by a psychiatrist (criteria 3). Information on this was taken by the electronic patient files noted by the ambulant urologist. Criteria 1 was proven by the ambulant urologist with the question: “Do you feel compromised in your well-being or body consciousness due to cosmetic or optical consequences caused by the operation?”.

## 3. Results

### 3.1. Patients' Demography

Average age of patients at the time of OE-R was 72.1 (54–91) years, and general condition of patients according to ECOG criteria was median 0 (*n* = 41; 49.4%). Altogether 17 patients (20.5%) showed an ECOG performance status of ≥2. In all cases, histologically affirmed adenocarcinoma of the prostate was present. 70% (*n* = 58) of patients had an undifferentiated tumor (solitary Gleason pattern: ≥4). In 67 patients, preoperative PSA value was known, and the median value was 144 ng/mL (IQR: 68–259). In 9.6% patients (*n* = 8), AD primarily resulted in achievement of maximum hormone blockade (OE-R plus antiandrogen).

### 3.2. Operation Parameter

In 62 patients (74.7%), OE-R was performed in local anesthesia. The remaining 21 patients additionally received sedoanalgesia. Representing a surgical procedure mostly performed by residents (*n* = 74), the operation time was 25 minutes (11–47 minutes) with a median of 20 minutes.

Altogether, 12 postoperative complications (14.4%) were described, which were managed conservatively in 7 cases (3x hematoma and 4x wound infection). In 5 patients, surgical revision (2x massive hematoma and 3x abscess) was necessary.

### 3.3. Oncological Followup

**Figure 1:**
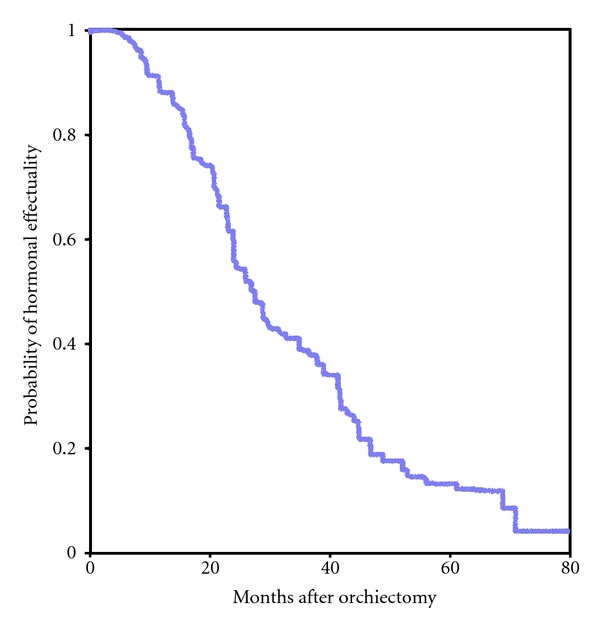
Time of tumor remission in 83 patients with osseous metastasized prostate cancer after subcapsular orchiectomy (Kaplan-Meier method).

Median time of hormonal efficacy was 29 months, whereby 11% of patients (*n* = 9) showed tumor remission until 5 years beginning from OE-R (Figure 1). Median overall survival of 36 months was analyzed. After 5 years, 16% (*n* = 13) of the patients were still alive. The mean PSA nadir achieved was 10.5 (0.01–212) ng/mL, but 45% patients (*n* = 37) achieved a PSA nadir under 2 ng/mL. Six patients (7.2%) showed immediate tumor progression after OE-R. Seven patients of the study group (8.4%) underwent chemotherapy for further treatment during castration-resistant status (different protocols, *n* = 4 with Mitoxantrone 12 mg/m² q3w).

During further disease progression, 12% of patients suffered from pathologic bone fractures (*n* = 10). According to electronic patient files received from the ambulant urologists, there was no patient with psychological problems (criteria 1–3, Section 2) concerning OE-R. More than one half of patients (*n* = 48, 58%) declared to suffer psychologically under general consequences of androgen withdrawal (disturbances, depression, tiredness, muscle wasting, osteoporosis, loss of libido, and erectile dysfunction).

## 4. Discussion

In 1895, White demonstrated the hormone sensibility of the prostate by treating 111 men with an obstructive prostate hyperplasia by surgical castration [10]. In 1935, David et al. succeeded in isolating testosterone. Subsequently, Huggins and Hodges inaugurated the androgen deprivation as targeted therapy in advanced PCa [2]. For several decades, surgical castration displayed the gold standard in metastasized PCa. Huggins received the Nobel Prize for Medicine and Physiology in 1966 in appreciation of his scholarly achievements. Coy et al. and Labrie et al. first synthesized potent LHRH analogues in 1973. This new substance class has effectively been applied since the 1980s as standard therapy in metastasized PCa [11, 12]. 

Equivalence of surgical and medical castration with regard to remission and overall survival rate has been verified sufficiently [13, 14]. Median time of tumor remission in the present study was 29 months, which was in the upper range of the corresponding expectation values for PCa patients with primary AD therapy in tumor stage D2 [15].

One advantage of orchiectomy is rapid effectiveness, with achievement of castration level between 3 to 12 hours postoperatively [16]. This is an extremely important factor for symptomatic patients (ostealgia, imminent fracture, and compression of the spinal cord). A compromise of the oncological safeness with OE-R is not a matter of concern due to potential residual of testicular parenchyma. Postsurgical testosterone and LH level is comparable with values after bilateral radical orchiectomy [17, 18].

A second advantage is the definitive approach of surgical castration with respect to good compliance of patients. A disadvantage might occur when intermittent androgen deprivation (IAD) would be indicated [19]. In the basic studies, Goldenberg et al. added this form of therapy hoping for improvement of quality of life during the therapy-free interval and altogether for a prolonged duration of treatment during hormone sensitive status [20]. A large clinical study comparing a continuous with intermittent AD (SWOG 9346) showed no difference in overall survival [21]. In a recent study (SEUG), there was no evidence of better survival or an improvement of quality of life for IAD in comparison to continuous AD [22].

In 14% of patients in the present study, surgical side effects occurred; however, in only 5 patients, surgical revision was necessary. This high postoperative complication rate in comparison to an international level might eventually be based on the fact that in our study, the OE-R was mainly performed by residents (89%). Orchiectomy and the use of LHRH analogues or GnRH antagonists did only show slight differences concerning other side effects [23]. Hot flushes, lack of drive, loss of libido, and erectile dysfunction are the major side effects of castration therapy which were reported by patients. In our study group, 58% of patients suffered from psychical illness. In the long-time period, in approximately 50% of patients, osteoporotic changes occurred. In case of that, there seems to be a rationale to apply simultaneous therapy with bisphosphonates or RANK-ligand-inhibitors (Denosumab) [24, 25]. Due to the fact that prostate cancer mainly metastasizes into bones, a prevention of pathologic fractures is expected by the use of this comedication. 

A psychological strain caused by loss of testicles was not detectable in our study group (in accordance with criteria 1–3). Regarding this, a representative study by the Zoladex Prostate Cancer Group Study was initiated which compared quality of life and psychological status of patients with medical versus surgical castration [26]. In this study, including 147 PCa patients at tumor stages D1-D2 (Zoladex 115 and orchiectomy 32), patients were able to individually choose the kind of therapy, and, finally, an advantage was observed in the Zoladex arm concerning quality of life and psychological status. Especially in the sector of body awareness, patients with surgical castration reported detriment [26]. However, there are also studies with best evidence which report no difference in “body image” and “quality of life” between both treatment arms [27]. In the “Prostate Cancer Outcomes Study,” there was significantly more gynecomastia (25% versus 10%) and a reduced general health condition (35% versus 28%) by treatment with LHRH analogues compared to patients with surgical orchiectomy [28]. Furthermore, patients taking LHRH analogues critically described to feel more often reminded of their treatment and their cancer disease. Due to this, they felt a negative impact in quality of life [28]. 

Concerning the general worse assigned body image of patients with orchiectomy, which is caused by loss of testicles, the paper of Issa et al. should be taken into consideration [29]. In this study on 88 orchiectomized patients pretreated by LHRH analogues (*n* = 52), the weight of testicles in study group 1 was compared with study group 2, comprising patients undergoing orchiectomy without medical pretreatment (*n* = 36). There was a significantly lower median weight of testicles in patients with pretreatment (7 versus 15 gr.; *P* < 0.001) [29]. In consideration of volume-protecting effects after the surgical method described by Riba (OE-R), general data concerning deprivation of body awareness in patients after orchiectomy in comparison to medical castrated patients have to be interpreted more cautiously. In one study, Chadwick et al. showed that approximately 50% of men with advanced prostate cancer would have chosen orchiectomy if they had been offered this as a therapeutic option [30]. Furthermore, there is an extremely insightful study of Mariani et al., which again documents that 70% of patients with free option of treatment would choose LHRH analogue treatment [31]. However, in case of 20% self-maintenance in therapy costs, only 24% of patients would favor medical hormonal ablation [31]. 

Each study assessing cost efficiency of therapy clearly affirmed an advantage for orchiectomy in comparison to medical castration. Even older studies, that are describing definitely longer periods of hospitalization after orchiectomy, agree with this statement [32, 33]. In the study of Mariani et al. which already has been quoted, 96 patients were analyzed. Treatment with LHRH analogues was assessed to be 10.7x to 13.5x more expensive than surgical castration [31]. Deliberations concerning treatment should include the limited financial valences of the public health system, considering that in case of equality of treatment effects, an inadequate resource policy would be short-sighted and, furthermore, limit treatment options for other patients. 

The present study shows some limitations, which have to be considered with regard to interpretation of the results. It is a retrospective study based on a limited number patients included in the study group (only 8 patients were added per year), who have been treated during a relatively long time span and by different surgeons. Period of examination (1990–2000) took place before the approval of Docetaxel; this might be one of the reasons for the worse median overall survival (35 months). Lacking availability of chemotherapy on the basis of evidence recommendation during the evaluation period, only 8% of patients were treated with chemotherapy at castration-resistant tumor stage (CRPC). Most patients only received best supportive care. In addition to that, only in 81% of patients, the preoperative PSA values were known. It has to be critically noted that the evaluation of psychological disorders, caused by loss of testicles, was not determined based on standardized and validated questionnaires. 

In summary, the present study shows that subcapsular bilateral orchiectomy described by Riba (OE-R) remains a very effective procedure with few side effects in the primary treatment of metastatic PCa. This procedure combines a high patient comfort with the absence of mental disorders and low costs for a financial limited public health system.

## 5. Conclusions

Against the background of cost explosion in the health care system, OE-R might experience a renaissance as an effective therapeutic option of androgen deprivation and as a cost-effective method with only few side effects. Hence, OE-R could regain an increasing relevance in the primary therapy of metastasized PCa.

##  Conflict of Interests

The authors report no conflict of interests. The authors alone are responsible for the content and the writing of the paper.

##  Authors' Contributions 

O. Rud and J. Peter prepared the data for statistical analysis and helped to draft the paper. R. Kheyri conceived the study, participated in its design and coordination, and carried out acquisition of data. C. Gilfrich and A. Ahmed supervised design and process of the study. W. Boeckmann and P. Fabricius participated in the design of the study and helped to draft the paper. M. May participated in the design and coordination of the study, carried out statistical analysis and interpretation of data, and drafted the paper. All authors read and approved the final paper. O. Rud, J. Peter, and R. Kheyri are contributed equally to this paper.
